# Sleep hygiene games and gamification: where are we and where are we heading?

**DOI:** 10.3389/frsle.2025.1607117

**Published:** 2025-07-30

**Authors:** Zilu Liang, Edward Melcer, Nhung Huyen Hoang, Kingkarn Khotchasing

**Affiliations:** ^1^Ubiquitous and Personal Computing Lab, Faculty of Engineering, Kyoto University of Advanced Science (KUAS), Kyoto, Japan; ^2^Institute of Industrial Science (IIS), The University of Tokyo, Bunkyo, Tokyo, Japan; ^3^School of Computer Science, Carleton University, Ottawa, ON, Canada

**Keywords:** sleep, serious games (SGs), sleep hygiene (SH), gamification (GAM), ubiquitous and mobile computing, mHealth (mobile Health), digital health

## Abstract

Sleep hygiene encompasses the habits, behaviors, and environmental adjustments conducive to achieving a restful sleep at night. Practices such as maintaining a consistent bedtime routine, avoiding sleep-disruptive substances such as alcohol and caffeine, and ensuring a dark and quiet bedroom are examples of good sleep hygiene. There is growing evidence that well-designed serious games can facilitate desired healthy behavioral changes, suggesting their potential for sleep hygiene intervention. This paper presents a narrative review of existing serious games and gamified systems designed for sleep hygiene intervention in daily life, providing an overview of the current landscape of Sleep Hygiene Games and Gamified Systems (SHG2S). We searched for peer-reviewed publications using four databases including Web of Science, PubMed, ACM Digital Library, and IEEE Xplore. The analysis focused on the targeted sleep hygiene, game design elements, and system evaluation and validation. We found that Accomplishment (e.g., rewards, level-up, leaderboard), Avoidance (e.g., punishment), and Social Relatedness (e.g., group quest, social prod, friending) were the most frequently employed game designs in SHG2S. Most SHG2S focused on addressing nighttime routine, while optimizing sleep environment has largely been underexplored. Existing SHG2S are also limited in addressing different cultural background and sleep patterns. Overall, empirical evidence is still limited regarding whether, why and how gamification leads to favorable effects on sleep hygiene over various temporal scales. Future work should focus on establishing a comprehensive and standardized evaluation framework to facilitate cross-study comparison and to collect evidence on the effectiveness of SHG2S.

## 1 Introduction

Adequate and high-quality sleep is vital for both mental and physical health (Reid et al., 2006; Clement-Carbonell et al., 2021; Ramar et al., 2021). However, sleep quality is heavily influenced by lifestyle choices and habits. Improving sleep, therefore, requires intentional behavior changes aimed at better sleep hygiene (Irish et al., 2015; Grandner, 2019; Liang et al., 2016; Liang, 2022). However, implementing and sustaining these changes over the long term can be challenging (Wilson et al., 2014; Rapp and Cena, 2014). To address this, innovative strategies such as gamification and serious games have emerged as powerful tools for promoting lasting behavior change (Connolly et al., 2012; Hamari et al., 2014; Boyle et al., 2016; Sardi et al., 2017; Chou, 2019).

Although seemingly similar, gamification and serious games differ in their approaches toward eliciting behavior change. Gamification integrates game features into non-game contexts (Deterding et al., 2011), aiming to enhance user experience or motivate behavior change (Seaborn and Fels, 2015; Alahäivälä and Oinas-Kukkonen, 2016; Chou, 2019). In health, gamification has been successfully applied to promote physical activity (Goh and Razikin, 2015; Johnson et al., 2016), encourage healthy eating (Lister et al., 2014), and support diabetes management (Theng et al., 2015; Priesterroth et al., 2019). Its low resource requirements make gamification ideal for mobile and wearable devices (Cechetti et al., 2017), allowing users to easily engage with mobile health interventions (Dennison et al., 2013; King et al., 2013).

On the other hand, serious games are specifically designed games with an additional “characterizing goal” beyond entertainment, such as education or health promotion (Dörner et al., 2016). They can lead to cognitive, behavioral, and motivational changes (Connolly et al., 2012; Boyle et al., 2016; Hamari and Keronen, 2017). In the context of health, serious games have been shown to increase engagement (Wattanasoontorn et al., 2013), drive motivation (Papastergiou, 2009), teach health knowledge (Primack et al., 2012; Lu and Kharrazi, 2018), improve adherence (Kato, 2010), elicit healthy behavior change (Haaranen et al., 2014; Smeddinck, 2016), and extend the reach of intervention programs to those who might not otherwise use them (Lenihan, 2012; Fleming et al., 2017).

Despite the success of gamification and serious games in promoting health behaviors, their application to sleep health is still in its early stages. Research emphasizes the importance of sleep hygiene for enhancing sleep health (Baranwal et al., 2023; Baron et al., 2021; Irish et al., 2015). Sleep hygiene comprises four key aspects (Irish et al., 2015; De Pasquale et al., 2024) establishing a consistent sleep-wake cycle (SH1), establishing a nighttime routine (SH2), optimizing the sleep environment (SH3), and cultivating healthy daily habits (SH4). This paper explores how games and gamification have been employed to address these aspects of sleep hygiene through research-grade Sleep Hygiene Games and Gamified Systems (SHG2S). We analyze SHG2S in terms of targeted sleep hygiene aspects, motivational design elements, and evaluation methods. Our findings offer an overview of the current state of SHG2S, discuss challenges, and highlight opportunities for future studies.

## 2 Materials and methods

This review aims to provide an overview of recent progress in sleep game research. We believe that such an overview is timely and can clarify current knowledge gaps and identify novel research opportunities at the intersection of sleep health and game research.

We followed a narrative review procedure (Sukhera, 2022) to identify published studies on SHG2S. Given the novelty of sleep game research, we restricted our search to studies between January 2018 to January 2025. We searched for four online databases (Web of Science, PubMed, ACM Digital Library and IEEE Xplore) using keywords: “Game or gamification or serious game or game-based,” “sleep or sleep hygiene.” We excluded terms such as “sensor or addition or videogame or excessive gaming or problematic gaming” to filter out studies on wireless sensor networks and the negative affects of entertainment video games on sleep. A total of 120 entries were retrieved. These were imported into Rayyan, a collaborative software for literature review. After removing three duplicates, three authors independently reviewed titles and abstracts based on inclusion criteria. Study had to (1) use gamification or serious games and (2) address sleep habits or behavior affecting sleep. We excluded studies on health conditions indirectly related to sleep (e.g., stress, anxiety) and apps that only monitor sleep data. Disagreements were resolved through discussion, leading to the inclusion of 5 publications at this stage. We also performed snowballing (Wohlin, 2014) by reviewing the reference lists of included papers and recent publications, which resulted in the inclusion of 4 more publications. In total, 9 publications presenting 13 SHG2S were imported into a spreadsheet for analysis. The analysis was guided by the Octalysis framework (Chou, 2019), a gamification design framework that lays out the eight core drives for human motivation.

## 3 Results

### 3.1 Targeted sleep hygiene

As shown in Table 1, 11 out of the 13 research-grade SHG2S were designed to address SH2, with four were exclusively designed for families with children (Shin et al., 2023; Pusateri et al., 2020, 2019; de Almondes and Leonardo, 2018). Six systems address SH1 and SH4, and only 2 focused on SH3. For example, Restful Journey includes educational content on maintaining bedroom temperature within an optimal range, while the Sleep Ninja app features an interactive “Bedroom Game” that prompts users to identify and click on items in a virtual bedroom that may disrupt sleep.

**Table 1:** Research-grade SHG2S included in the analysis.

**System name**	**Main concept**	**Target users**	**Target sleep hygiene**	**Game design elements**	**Validation study**
Perfect bedroom (de Almondes and Leonardo, 2018)	A tangible educational card game kit that requires players to build an ideal sleep environment	Children (7–9 years)	SH2	Accomplishment, avoidance, ownership, social relatedness	None (RCT planned)
Lights out (Pusateri et al., 2019)	A tangible system with lights and cards to help children establish a nighttime routine together with their family members	Children (3–5 years)	SH2	Accomplishment, social relatedness	None
Dream on (Pusateri et al., 2020)	A tangible choose-your-own-adventure aroma book that helps people fall asleep	Adults who sleep in unfamiliar environment	SH2	Empowerment, unpredictability	Field trial (*n* = 6)
Office shot (Pusateri et al., 2020)	A tangible social game that uses a token system to improve awareness of caffeine consumption at workplace	Sedentary office workers who consume caffeine	SH4	Social relatedness	Field trial (*n* = 2 groups)
Wander (Cai et al., 2021)	A mobile-app based breath-control audio game for reducing smartphone use before bed	Anyone	SH2	Accomplishment, unpredictability, social relatedness	None
Sleepy bird (Ilhan et al., 2022)	A mobile-app based serious game to enhance sleep-wake behavior	People with difficulty waking up	SH1	Accomplishment, avoidance	RCT (*n* = 26)
Bedtime pals (Shin et al., 2023)	A mobile app that allows parents to set bedtime routines for their child(ren) and to reward compliance	Children	SH2	Accomplishment	Field trial (*n* = 7 households)
Caring heart (Shin et al., 2023)	A tangible lighting system with a companion app that allows family to establish an ideal bedtime routine as a team	Children	SH2	Accomplishment	Field trial (*n* = 5 households)
Sleep Ninja (Werner-Seidler et al., 2023)	A mobile app that provides CBT-I training sessions to help develop healthy sleep habits and improve sleep quality.	Teenagers (12–16 years)	SH1, SH2, SH3, SH4	Accomplishment	RCT (*n* = 264)
Restful journey (Seaver et al., 2024)	A mobile app that teaches sleep hygiene and apply the knowledge in play scenarios	Adults with poor sleep (PSQI ≤ 5)	SH1, SH2, SH3, SH4	Accomplishment, unpredictability, empowerment	Field trial (*n* = 35)
Hero's Sleep Journey (Liang et al., 2024)	A smartwatch app that engages users in sleep hygiene practices by fighting monsters	University students	SH1, SH2, SH4	Accomplishment, empowerment	None
Sleep Tamagochi (Liang et al., 2024)	A smartwatch app that engages users in sleep hygiene practices by raising virtual pets	University students	SH1, SH2, SH4	Accomplishment, ownership, social relatedness	None
Sleepland (Liang et al., 2024)	A smartwatch app that engages users in sleep hygiene practices by building a village	University students	SH1, SH2, SH4	Accomplishment, ownership	None

### 3.2 Game design elements

Accomplishment is the most frequently employed motivational drive in SHG2S. Most systems incorporate extrinsic motivation, such as bonus cards and leaderboard in Perfect Bedroom (de Almondes and Leonardo, 2018), avatar level-ups in Sleep Ninja (Werner-Seidler et al., 2023), and smart lights as progress bars in Lights Out (Pusateri et al., 2019). Wander (Cai et al., 2021) rewards users with clearer morning images if they spend less time on their phones the night before. These game features motivate users by fostering a sense of growth and satisfaction.

The Avoidance dimension of the Octalysis framework serves as another extrinsic motivator, tapping into the fear of losing something or being punished. Sleepy Birds (Ilhan et al., 2022) employs complex punishment rules to discourage non-compliance with the planned sleep-wake schedule. For example, users lose one life for each 5 min of sleeping past the ideal wake-up time. The bird moves faster if users sleeps too early or too late, making it harder to control the bird's movement. Furthermore, users fall behind in competition by 25 meters for each 5 min of snoozing. Similarly, Perfect Bedroom (de Almondes and Leonardo, 2018) implements punishment through “pass the turn” cards that slow players' progress if they exhibit poor sleep hygiene. Notably, the Sleepy Birds study demonstrated that employing avoidance in the design of SHG2S led to better outcomes. Compared to a non-gamified version of the system, the game version motivated users to snooze less and wake up closer to the planned time to avoid losing lives in the game (Ilhan et al., 2022).

Social Relatedness is another key motivational drive used to boost users' intrinsic motivation. People often make sleep-related decisions within their social context, and prior studies have highlighted sleep games as a social practice (Pusateri et al., 2020). For example, Lights Out (Pusateri et al., 2019), Bedtime Pals (Shin et al., 2023), and Caring Heart (Shin et al., 2023) were designed for families with children, engaging both parents and children to work together to establish a pre-bed routine, thereby improving sleep for the entire family. Wander (Cai et al., 2021) allows users to share information with friends to collaboratively unravel the mystery of the Mist Forest. Office Shot (Pusateri et al., 2020) employs group quests to promote mindfulness of coffee consumption among office workers. Social features can be designed to encourage both competition and collaboration. For example, while Sleep Tamagochi allows users to play together with friends (Liang et al., 2024), Perfect Bedroom (de Almondes and Leonardo, 2018) enable users to battle with one another, motivating them to maintain good sleep hygiene to outperform their peers. Empirical evaluation show positive outcomes. The Office Shot became a talking point among coworkers to discuss sleep (Pusateri et al., 2020), while both Lights Out and Caring Heart led to positive behavior change in children before and during sleep (Pusateri et al., 2019; Shin et al., 2023).

In addition to social relatedness, humans also have an inherent need for Empowerment and Creativity. People enjoy games for the positive emotions and creativity they foster. Several SHG2S incorporate design elements surrounding Empowerment and Scarcity to create an engaging and playful experience. For instance, Restful Journey (Seaver et al., 2024) incentivizes players with earned coins to explore various islands and collecting jewels. Another way to engage players is by adding unpredictability to the game. Restful Journey (Seaver et al., 2024) introduces a random sleep goal for player to achieve. The Dream On aroma book (Pusateri et al., 2020) elegantly combines Empowerment and Unpredictability to enhance the playfulness of the game. Users are given tokens with different aroma scents to choose from, and their choices influence how the story progresses. In essence, players co-create a bedtime story with the system based on their lived experience of that moment.

### 3.3 Evaluation and validation

Evaluating SHG2S requires assessing their playability, usability, effectiveness in inducing behavior change, and their impact on sleep quality. Our analysis revealed that only about half of the games were empirically evaluated, typically over short periods of two weeks (Seaver et al., 2024; Ilhan et al., 2022; Pusateri et al., 2020; Shin et al., 2023). Sleep Ninja conducted the largest study, a randomized controlled trial (RCT) with 264 subjects, showing significant reduction in insomnia symptoms at 6 and 14 weeks with medium effect sizes (Werner-Seidler et al., 2023). The Sleepy Birds study (Ilhan et al., 2022), with a smaller sample (*n* = 26), used RCT design to compare the game version of the app with a non-game version. Results indicated that the game version helped users snooze less and adhere more closely to their sleep schedule, with a post-use questionnaire showing that 85% of participants found the app enjoyable. The Restful Journey app underwent a before-after comparison with 35 subjects with poor sleep, showing statistically significant improvement in sleep quality and anxiety levels over a 30-day field trial. The study found effect sizes ranging from small to large, although it lacked a control group (Seaver et al., 2024).

Dream On (Pusateri et al., 2020), Office Shot (Pusateri et al., 2020), Bedtime Pals (Shin et al., 2023), and Caring Heart (Shin et al., 2023) were evaluated through field trials with small cohorts (*n* = 2–7). Semi-structured interviews revealed positive impact on pre-sleep arousal and stimulated a sense of control in an unfamiliar sleep environment. Dream On participants reported that the sensory input helped reduce pre-sleep anxiety, while Office Shot participants showed improved awareness of sleep-related choices (Pusateri et al., 2020). Bedtime Pals and Caring Heart were positively received by children and parents, though they did not result in substantial sleep quality improvements for the parents (Shin et al., 2023).

## 4 Discussion

### 4.1 Principal findings

Among the motivational drives in SHG2S, Accomplishment (e.g., progress bars, level-ups, leaderboards) is the most commonly used. This aligns with prior research on gamified health systems (Chou, 2019; Stepanovic and Mettler, 2018). Other frequently used elements include Avoidance (e.g., punishment) and Social Relatedness (e.g., group quest, social prod, friending), followed by Ownership and Empowerment. In contrast, Epic Meaning & Calling is notably absent in all analyzed systems. This presents a promising design opportunity: future SHG2S, especially those aimed at families or couples, could integrate shared quests or missions that tap into users' sense of purpose, such as enhancing family wellbeing or nurturing a supportive relationship. Framing sleep health as a collaborative journey toward mutual goals may strengthen user motivation and promote sustained engagement.

While surface-level elements such as points and leaderboards can provide short-term motivation, their impact tends to diminish over time (Stepanovic and Mettler, 2018; Zuckerman and Gal-Oz, 2014), and excessive external rewards can undermine intrinsic motivation (Deci et al., 1999). Some SHG2S, such as Perfect Bedroom (de Almondes and Leonardo, 2018), Lights Out (Pusateri et al., 2019), Wander (Cai et al., 2021), combine Accomplishment and Social Relatedness to promote both extrinsic and intrinsic motivation. However, motivational drives like Empowerment, Social Influence, and Unpredictability/Curiosity are underutilized and could further enhance long-term engagement.

Our analysis also revealed that current SHG2S mainly target non-clinical populations in Western countries, focusing on sleep regularity (SH1) and nighttime routines (SH2). This excludes individuals with sleep disorders such as insomnia or sleep apnea. Furthermore, important aspects of sleep hygiene, such as optimizing the sleep environment (SH3) and promoting healthy daytime habits (SH4), are often neglected. A notable strength of existing SHG2S, however, is their occasional focus on children and families. These systems often feature tactile, multi-sensory games that can be played in various areas of the home, such as bedrooms or living rooms.

Sleep games as a research field is still in its infancy. Currently, empirical evidence is limited as to whether, why, and how gamification leads to favorable effects on sleep hygiene across different temporal scales. There could be two reasons for this gap. First, SHG2S are inherent complex, aiming not only to change user behaviors but also to improve the multifaceted phenomenon of sleep, which involves various interconnected factors (Baranwal et al., 2023; Jackson et al., 2020; Irish et al., 2015). For instance, interventions targeting sleep environment optimization (SH3) differ from those promoting healthy exercise and nutrition habits (SH4). The introduction of game elements further complicates evaluation, as it requires balancing behavior change with creating engaging and enjoyable user experience. Second, evaluating the effectiveness of SHG2S is challenging because the effects of sleep hygiene interventions take time to manifest in sleep quality (Baron et al., 2021). Many studies on SHG2S have been short-term (e.g., 2 weeks) (Pusateri et al., 2020; Shin et al., 2023), which may not be sufficient to observe significant improvement in sleep quality. As a result, much of the research to date has focused on usability and user satisfaction rather than measurable sleep improvements. Given the heterogeneity of game components, target users, and usage scenarios, direct comparison and synthesis across studies remain difficult.

### 4.2 Challenges and opportunities

To broaden the impact of SHG2S, future designs should address key gaps in the current systems. First, a more comprehensive use of diverse motivational drives in game design is essential. Elements like social support, feedback, and sharing can enhance user engagement, as demonstrated in other gamified health apps (Johnson et al., 2016). In addition, introducing variability and uncertainty in rewards has proven effective in boosting engagement in health apps (El-Hilly et al., 2016). Second, future SHG2S designs should target a broader demographic, including teenagers, college students, and senior citizens. It is also important to address common sleep disorders while incorporating under-explored aspects of sleep hygiene, such as optimizing the sleep environment (SH3) and promoting healthy daytime habits (SH4). Contextualizing sleep within the entire daily cycle may present new opportunities for SHG2S to influence daytime behaviors that, in turn, improve sleep quality (Liang et al., 2025). Finally, a robust and standardized evaluation framework is essential for advancing SHG2S research. This framework should encompass playability, usability, emotional impact, user attitudes, and efficacy in improving sleep quality. By integrating these elements, researchers will be better equipped to assess the true benefits of SHG2S in enhancing sleep hygiene and quality. Moreover, a holistic evaluation approach will enable more meaningful cross-study comparisons and strengthen the evidence for SHG2S effectiveness and drive progress in the field.
